# Supplementary material for: FGF1 Protects MCF-7 Cells against Taltobulin through Both the MEKs/ERKs and PI3K/AKT Signaling Pathway
Source: Biomedicines. 2023 Jun 29;11(7):1856. doi: 10.3390/biomedicines11071856 (PMC10376943; doi:10.3390/biomedicines11071856)
Supplement: Supplementary file 1 [file biomedicines-11-01856-s001.zip › biomedicines-2398237-supplementary.pdf]

SUPPLEMENTARY DATA

SUPPLEMENTARY FIGURE 1

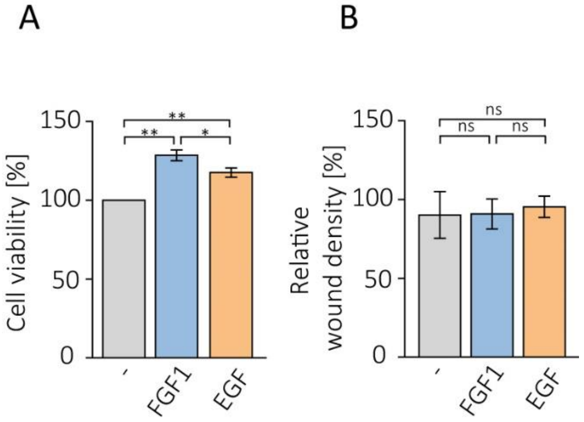

Supplementary Figure S1. Effects of FGF1 and EGF on MCF-7 cell proliferation and migration. (**A**) Effects of FGF1 and EGF stimulation (10 ng/mL, 48 h) on cell proliferation in the absence of cytotoxic drugs **(B)** The effect of FGF1 and EGF stimulation (10 ng/mL, 36 h) on cell migration in the absence of cytotoxic drugs. Data were normalized and are presented as mean values ± standard deviation (SD) from three independent experiments. Statistical significance: * $p < 0.05$, ** $p < 0.01$, no significant differences indicated as 'ns'

SUPPLEMENTARY FIGURE 2

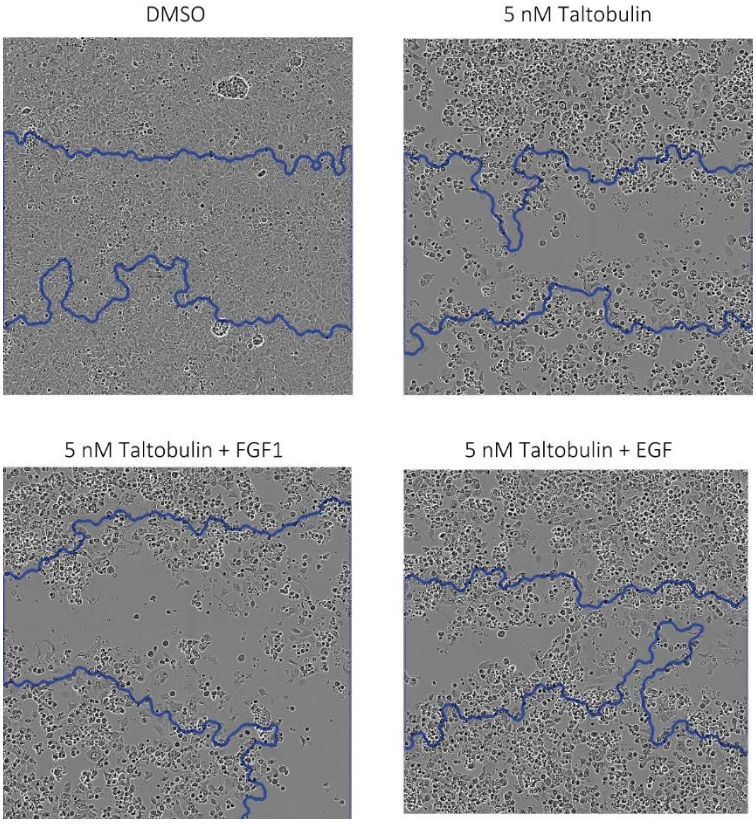

Supplementary Figure S2. Effects of FGF1 and EGF on MCF-7 cell migration in the presence of taltobulin. The effect of FGF1 and EGF on MCF-7 cell migration in the presence of 5 nM taltobulin was examined after 36 h using IncuCyte® Cell Migration and Invasion System. Presented data are representative images of wound healing.
